# Cancer and Thrombosis: New Treatments, New Challenges

**DOI:** 10.3390/medsci9020041

**Published:** 2021-06-03

**Authors:** Anders Erik Astrup Dahm

**Affiliations:** 1 Department of Hematology, Akershus University Hospital, 1478 Lørenskog, Norway; aeadahm@gmail.com; 2 Institute of Clinical Medicine, University of Oslo, 0316 Oslo, Norway

**Keywords:** cancer, thrombosis, treatment, prophylaxis, anticoagulation

## Abstract

The direct-acting oral anticoagulant (DOAC) has become an alternative to low-molecular-weight heparin (LMWH) for treatment and prophylaxis of venous thromboembolism (VTE) in cancer patients. The clinicians are, however, faced with difficult decisions regarding DOAC treatment: Which patients cannot use DOACs? Should incidental VTE be treated similar to symptomatic VTE? Is it safe to give DOACs to patients with gastrointestinal or urogenital cancers? How about drug–drug interactions? Should all cancer patients receive thromboprophylaxis? Is arterial thrombosis a problem? The current article reviews the available literature regarding these questions and aims to provide practical solutions based on data from the clinical trials and new guidelines.

## 1. Introduction

The first known observation of the association between cancer and venous thromboembolism (VTE) is attributed to Jean-Baptiste Bouillaud [1]. In 1865 Armand Trousseau described this association in more detail in his chapter “Phlegmasia alba dolens” from the published collections of his lectures [2]. VTE in cancer patients is a major complicating factor for cancer patients, and is associated with increased mortality [3]. One study found that venous and arterial thromboembolism accounted for 9.2% of deaths in cancer patients [4]. In the last two decades, the knowledge of treatment and prophylaxis of cancer-associated thrombosis has increased substantially. The introduction of the direct-acting oral anticoagulants (DOAC) has resulted in more treatment options, but also more complicated decisions. The term “cancer-associated thrombosis” usually refers to VTE, but arterial thrombosis in cancer patients may be a problem that deserves more attention than it receives. Bleeding is the main side-effect of anticoagulant therapy. Cancer patients are more prone to bleeding than other patients for different reasons, e.g., thrombocytopenia, growth of the solid cancers into internal organs, or the development of disseminated intravascular coagulation. Hence, in anticoagulant treatment of cancer patients it is pivotal to weigh the risk of thrombosis against the risk of bleeding. Recently, several organizations have published guidelines for treatment and prophylaxis of VTE in cancer patients [5,6,7].

## 2. Epidemiology of Venous Thrombosis in Cancer Patients

It is difficult to find precise estimates of the incidence of VTE in cancer patients. The main reason is that the frequency of VTE varies between cancer patients and depends on cancer type, treatment (e.g., surgery, conventional chemotherapy, anti-estrogens), complications (e.g., hospitalization, infections, dehydration, immobilization), and each patient’s personal risk for VTE (e.g., familial thrombosis, previous thrombosis). In a registry study from the USA of patients receiving chemotherapy, 13.5% had VTE within 12 months [8]. The cancers investigated were from the lung, colon, ovary, bladder, stomach and pancreas. In a more recent registry study from the UK, the authors estimated the incidence of VTE in patients with active cancer, defined as a primary diagnosis of cancer or treatment for cancer within a 180-day period [9]. They included a wide range of common cancers. The incidence rates they found are given in Table 1 and show that cancers of the lung, brain, ovary, pancreas and stomach have an incidence of first VTE of 10–15% per year, while other cancer types have an incidence of 3–5% per year. For the clinician, however, the frequency of VTE in patients with breast and prostate cancer may appear high because these cancers are more common than, e.g., pancreas cancer.

Another interesting observation from this study was that the incidence rate of recurrent VTE in cancer patients peaked the first 180 days after the first VTE with 22 per 100 person-years and decreased over the next five years to 2.5 per 100 person-years.

VTE risk varies with the phases of cancer treatment. It is highest just before and right after the cancer diagnosis [10]. If the patient needs surgery, the risk will increase even further. The risk of VTE is reduced if the cancer is cured or in remission, and then increases when the cancer recurs [11].

## 3. Treatment

### 3.1. The Change from Vitamin K Antagonists to Low-Molecular-Weight Heparins

Several randomized studies comparing vitamin K antagonists with low-molecular-weight heparins (LMWH) for treatment of VTE in cancer patients have been conducted [12,13,14,15,16]. Collectively, these studies show that LMWH results in fewer recurrent VTEs than vitamin K antagonists, with approximately the same number of major bleedings. Most important among these studies is the CLOT study, which changed treatment from Vitamin K antagonists to LMWH when it was published in 2003 [13]. The CLOT study randomized 676 cancer patients with symptomatic VTE between coumarin and the LMWH dalteparin for 6 months treatment. Target INR for coumarin treatment was 2.0–3.0, while dalteparin was given as 200 IU/kg once daily for one month, then 150 IU/kg once daily for five months. The results showed that treatment with dalteparin resulted in fewer recurrent VTEs than coumarins, while the number of major bleedings was almost the same. “Major bleeding” was defined as a fall in hemoglobin of 2 g/dL or more, transfusion of two or more units of packed red blood cells or whole blood, occurring in a critical site (intracranial, intraspinal, intraocular, pericardial, intra-articular, intramuscular with compartment syndrome, or retroperitoneal), or contributing to death. This definition of major bleeding has been used in all the important studies on cancer-associated VTE since then. The CLOT study also showed that 40% of the patients died during 6 months of treatment, where 90% of the deaths were due to progressive cancer.

How good is LMWH in treating VTE in cancer patients? The Daltecan study, published in 2015, when LMWH had been standard therapy for cancer-associated VTE for several years, provides some insight. It was a single-armed interventional study of 334 cancer patients treated for VTE with dalteparin 200 IU/kg once daily for 4 weeks followed by 150 IU/kg from 2–12 months. During 12 months treatment, 10% had major bleedings and 11% recurrent VTE, but about a third of the major bleedings and half of the recurrent VTEs occurred during the first month of treatment. During the last 6 months, the frequency of VTE and major bleedings was lower than during the first 6 months of treatment.

Table 2 gives an overview of the most important studies on vitamin K antagonists and LMWH before the direct-acting oral anticoagulants (DOAC) were available. A simple pooled estimate of the frequency of recurrent VTE and major bleeding during 6 months of treatment with LMWH from these studies shows 7.6% recurrent VTE and 5.1% major bleedings [13,15,16,17].

### 3.2. Studies on Direct-Acting Oral Anticoagulants in Cancer Patients

After the publication of the studies of DOAC treatment for VTE in the general population, it was immediately clear that there was not enough evidence on cancer patients. The two main reasons were that the few cancer patients included in those phase III studies were not representative for the cancer population, and the comparator was vitamin K antagonists, which was not standard treatment for cancer patients. From 2017, several clinical trials on DOAC treatment of cancer-associated VTE have been published (Table 3). The conclusion from all the large pharmaceutical industry sponsored randomized trials is that the DOACs edoxaban, rivaroxaban and apixaban are non-inferior to the LMWH dalteparin in treatment of cancer-associated VTE. The tendency for the studies was, however, that DOAC treatment gave somewhat more bleedings and somewhat less recurrent VTE, with a possible exception for the studies on apixaban.

### 3.3. Which Cancer Patients Should Not Receive DOACs?

The possibility of DOAC treatment of cancer patients makes it even more important to evaluate the patient individually and be aware of which patients that were not included in the clinical trials (Table 4). All the interventional studies on DOAC treatment for cancer-associated VTE except the Cap study excluded patients with Eastern Cooperative Oncology Group status (ECOG) 3 and 4. The Hokusai VTE Cancer study and the Caravaggio study also had short life-expectancy as an exclusion criterion. Thus, DOAC have mainly been tested in more healthy cancer patients. Furthermore, the Hokusai VTE Cancer study, the Select-D study, and the Caravaggio study did not include other VTEs than deep-vein thrombosis or pulmonary embolism, while the Adam-VTE and Cap study included all kinds of VTEs, including thromboses of the splanchnic, cerebral, and upper extremity veins. Another important difference is that the Caravaggio study did not include patients with brain cancers or intracerebral metastases. None of the studies included patients with platelet count below 50 × 10^9^/L or creatinine clearance < 30 mL/min. Thus, DOAC treatment can be considered for all types of VTE, but the data are best for deep-vein thrombosis of the legs and pulmonary embolism. An important limitation is of course that patients must be able to take and absorb the tablets.

### 3.4. DOAC and Major Bleedings in Patients with Gastrointestinal and Genitourinary Cancer

An important observation from the clinical trials of edoxaban and rivaroxaban was that patients with gastrointestinal cancer, and maybe also genitourinary cancer, had more major bleedings on DOAC than on LMWH. The increased frequency of major bleedings appeared to be evident for all forms of gastrointestinal cancer [24]. Table 5 shows the frequency of major bleedings among patients with gastrointestinal and genitourinary cancers in the studies reporting these numbers. As one can see, the numbers of major bleedings are very low for genitourinary cancers, which makes the estimates very uncertain. Regarding gastrointestinal cancer, the most convincing data suggesting more major bleedings in patients receiving DOAC compared with dalteparin comes from the study on edoxaban [24]. The study on rivaroxaban also suggests increased risk of major bleedings among patients with gastrointestinal cancer, but the numbers from this study are influenced by the stopped inclusion of patients with cancer of the esophagus or gastroesophageal junction [20]. On the other hand, none of the studies on apixaban show any increased major bleedings in patients with gastrointestinal cancer. The Cap study did not have a control arm with dalteparin, but the frequency of major bleedings was no higher in gastrointestinal cancer than in other cancers [23,25].

An interesting observation from the Caravaggio study was that only patients with unresected gastrointestinal cancer had major bleeding in both study arms [25]. This suggests that patients with resected gastrointestinal cancer do not have increased risk of bleeding. Such data has not been published for rivaroxaban and edoxaban.

### 3.5. Should Incidental VTE Be Treated in Cancer Patients?

The problem with incidental VTE discovered on computer tomography (CT), in particular incidental subsegmental pulmonary embolism, is that it may be a false finding, because in the CT protocols used to diagnose pulmonary embolism the pictures are taken when the contrast is at its peak in the pulmonary arteries. For conventional thoracic CTs used to assess cancer, however, the pictures are taken when there is less contrast in the pulmonary arteries, and thus the diagnostic accuracy is lower. Moreover, since evaluation of cancer treatment is regularly done with computer tomography, incidental VTE is common in cancer patients. If an incidental VTE is discovered, it is recommended to carefully review the patient history to exclude symptomatic VTE. Cancer patients, and their treating physicians, may interpret symptoms of e.g., pulmonary embolism as expected fatigue [26]. It is suggested that if it is seen more than one incidental subsegmental pulmonary embolus, or if it is seen in larger pulmonary arteries, it is considered a true pulmonary embolism. Is there only one subsegmental pulmonary embolus, it is suggested to do additional investigations of the veins of the legs [27].

The studies on cancer-associated VTE investigating vitamin K antagonists and LMWH did not include incidentally discovered VTEs. A prospective observational study, where most patients were treated with LMWH, found 6% recurrent VTE during 12 months of treatment for incidental VTE [28]. The DOAC trials included from 20% to 54% incidental VTEs. Table 6 shows that the rates of recurrent VTE were somewhat higher in the patients with symptomatic VTE than in those with incidental VTE in the Hokusai VTE cancer study and in the Caravaggio study. This was also found in the Select-D study (only hazard ratios given), but not in the Cap study. Although some of the VTEs diagnosed incidentally were probably not true, the recurrence rate in patients with incidental VTE is high enough to justify anticoagulant treatment in the same way as symptomatic VTE.

### 3.6. VTE at Unusual Sites

Thrombosis in the veins of the upper extremities, abdomen and brain, were only included in the two academic studies Adam-VTE and Cap [21,23]. The Adam-VTE study included 39 VTEs at unusual sites, but reported no data on the recurrence rate for this subgroup of VTEs. In any case, they only found one recurrent VTE in the apixaban arm. The Cap study included 32 patients with VTE at unusual sites, and had three recurrent VTEs and one major bleeding in this subgroup (personal communication). Thus, the data on VTE at unusual sites in cancer patients are too limited to decide if they are at higher or lower risk of recurrent VTE. The lack of data speaks in favor of a conservative approach, and treat such VTEs in the same way as other VTEs.

### 3.7. Treatment Length and Reduced Dose DOAC after 3–6 Months

Most guidelines suggest a minimum of 3–6 months of treatment for VTE in cancer patients. All the major studies on cancer-associated VTE have however investigated 6 months treatment. Thus, 6 months appears to be the best documented minimum treatment in cancer patients. If patients are not cured for the cancer or are still under treatment, it is recommended to treat patients as long as they have active cancer.

Many patients without cancer treated for VTE with apixaban or rivaroxaban reduce the dose after 3–6 months of treatment. This is based on the Amplify-Ext study and the Einstein-Choice study [29,30]. In the non-cancer population with a medium risk of recurrent VTE, reduced doses appear effective and safe. The reduced-dose treatment has never been tested in patients with cancer. The risk of recurrent VTE in cancer patients has not been studied in detail, but is assumed to be related to the aggressiveness of the cancer, whether the cancer disease is in remission or not, and the type of cancer treatment. Hence, without evidence of treatment with reduced dose DOAC in cancer patients, no guidelines are currently recommending low-dose DOAC as treatment for VTE in cancer, except when the dose is adjusted for renal function and interacting drugs.

### 3.8. The Role of LMWH in Cancer Patients with VTE

LMWH will probably be a useful alternative for almost all patients with cancer-associated VTE during short periods of their cancer treatment. The advantage of LMWHs compared with DOACs is the shorter halftime and the easiness of dose adjustment. Situations where this is an advantage could be, e.g., in relation to surgery or biopsy, expected thrombocytopenia, reduced renal function or increased risk of bleeding. The three DOACs discussed here are all mostly absorbed in the stomach and/or the proximal small intestine. Rivaroxaban is best absorbed with food [31]. Thus, patients who cannot take or do not absorb oral medication will need to use parenteral anticoagulation, where LMWH will be the natural first choice.

## 4. Drug Interactions with DOACs

Edoxaban, rivaroxaban, and apixaban are metabolized by cytochrome CYP3A4 and other enzymes and are substrates to P-glycoprotein. This means that strong inducers and inhibitors of these enzymes will influence the concentration of the drugs, and thereby, potentially increase the risk for recurrent VTE or bleedings.

Most of the knowledge about drug interactions regarding DOACs come from in vitro studies. It is, however, essential to obtain drug–drug interaction data from patients since the predicted interaction do not always result in the expected outcome. An example is a large observational study which investigated the effect of drug–drug interactions on major bleedings with dabigatran, rivaroxaban and apixaban in patients with atrial fibrillation in Taiwan [32]. They found that amiodarone, fluconazole, rifampicin and phenytoin increased the risk of major bleeding, while atorvastatin, digoxin, erythromycin/clarithromycin reduced the risk. This was surprising, since phenytoin and rifampicin are inducers of CYP3A4 and P-glycoprotein, and thus, the expected effect would be fewer bleedings. Whether this result was due to study design or unknown biological mechanisms is not clear. Another study investigated amiodarone, dronedarone, diltiazem, verapamil in patients using rivaroxaban or apixaban for atrial fibrillation [33], and compared with DOAC users who did not use any of the interacting drugs. They found an increased risk of bleeding in the group using interacting drugs (hazard ratio 1.8), but most of the bleedings were clinically relevant non-major bleedings or minor bleedings. Pham et al. investigated verapamil and diltiazem interaction with dabigatran, rivaroxaban, or apixaban [34]. They found a 40–50% increased risk of bleeding compared with patients who did not use interacting drugs. A recent publication from the Caravaggio study investigating recurrent VTE, major bleedings and death in patients using and not using anticancer agents found no indication of drug–drug interaction with apixaban [35].

Only a few drugs relevant for cancer patients are known to be strong inducers or inhibitors of CYP3A4/P-glycoprotein. Examples of such drugs are listed in Table 7. There are a large number of drugs with moderate, weak or unknown potential for interaction with DOACs used by cancer patients. Examples of such drugs include some antiemetic drugs, glucocorticoids, tyrosine kinase inhibitors, erythromycin, verapamil, grapefruit juice and cyclosporine. There are unfortunately little data on drug–drug interaction between such drugs and DOACs in cancer patients.

## 5. Prophylaxis

The field of thromboprophylaxis is difficult in all patients. One of the reasons is that tolerable level of VTE is set arbitrary and the recommendations vary between patient groups. Regarding pharmacological thromboprophylaxis, the risk of bleeding has to be weighed against the risk of VTE. The Protecht study and the Save-Onco study tested the LMWHs nadroparin and semuloparin as thromboprophylaxis in a general oncological outpatient population [29,36]. Both studies found that the frequency of VTE in the placebo group was about 3% and that LMWH approximately halved the frequency of VTE. Based on these studies, the numbers it was necessary to treat were considered too high to recommend VTE prophylaxis to all cancer patients (Table 8). Hence, when thromboprophylaxis was tested with the DOACs rivaroxaban and apixaban both studies used the Khorana risk score ≥ 2 to select patients at high risk of VTE for inclusion [37,38]. Consequently, the number needed to treat was reduced, although the relative reduction of VTE was again approximately half. Therefore, current guidelines state that it is necessary to estimate the risk of VTE in cancer patients to decide on thromboprophylaxis [7]. The best validated risk assessment tool is the Khorana score [39], but there are many other suggested scores such as, e.g., the Protecht score or the Vienna Cats score [40,41]. The Khorana score is based on the type of cancer, body-mass index, and hemoglobin, leukocytes, thrombocytes measured in blood. It is, however, important to remember that the risk assessment tools do not assess the risk of bleeding, which needs to be done by the clinician. Low-dose LMWH or low-dose apixaban/rivaroxaban can both be used as pharmacological prophylaxis. Currently, it is not known whether DOAC are better, worse or non-inferior to LMWH.

Although there is little data on thromboprophylaxis in hospitalized cancer patients, there is general agreement that all cancer patients submitted to hospital for a disease other than the cancer, e.g., infection, should be considered for thromboprophylaxis. Additionally, all cancer patients undergoing surgery should be considered for postoperative thromboprophylaxis if the bleeding risk is not very high [7].

## 6. Arterial Thrombosis in Cancer Patients

### 6.1. Epidemiology of Arterial Thrombosis in Cancer Patients

Arterial thrombosis in cancer patients has received less attention than venous thrombosis. A recent review of observational studies investigating arterial thrombosis in cancer patients concluded that cancer patients are at increased risk of arterial thrombosis, i.e., myocardial infarction and ischemic stroke. The risk is highest during the first months after the cancer diagnosis and in patients with pancreatic and pulmonary cancer [42]. Wang et al. investigated the temporal relationship between cancer and arterial thrombosis in an electronic health record database from USA. They found that cancer patients had the highest risk of arterial thrombosis in the first 30 days following cancer diagnosis [43]. Grilz et al. investigated a cohort of 1880 cancer patients with a median follow-up of 24 months and found 2.6% arterial thrombosis. During the same time period, 8.4% developed VTE [44]. The same group also reported that cancer patients had about 7-fold risk of arterial thrombosis compared with non-cancer patients based on register data from Austria [45]. A registry study from USA found that the 6-month cumulative incidence of arterial thromboembolism was 4.7% compared with 2.2% in a control population without cancer [46]. A study from the RIETE registry on VTE identified 5717 patients with active cancer and VTE where 1.1% developed arterial thrombosis during a median anticoagulation time of 7.3 months [47]. Strongman et al. investigated cancer survivors more than 1 year after cancer diagnosis and found increased risk of arterial thrombosis for many different cancer types [48]. Hence, there is no doubt that cancer increases the risk of arterial thrombosis. What is lacking is detailed information from prospective studies, more details on specific cancers, and effect of anticoagulation and prophylaxis for arterial thrombosis.

### 6.2. Anticoagulation and Protection against Arterial Thrombosis in Cancer Patients

Aspirin is given as thromboprophylaxis to cancer patients on special indications, e.g., multiple myeloma treated with thalidomide/lenalidomide/pomalidomide or to patients with myeloproliferative diseases. Otherwise, there is little focus on preventing arterial thrombosis in cancer patients. There are no studies investigating prophylaxis with platelet inhibitors against arterial thrombosis in the general cancer population. Some of the studies investigating anticoagulant prophylaxis or treatment have, however, reported both arterial and venous thrombosis as endpoints (Table 9). The studies Protecht and Cassini tested nadroparin and rivaroxaban as primary prophylaxis in cancer patients and found very low frequency of arterial thrombosis, which may have been reduced with nadroparin/rivaroxaban, but the numbers are too low to be conclusive [36,38]. In the Adam-VTE study testing apixaban versus dalteparin for VTE in cancer patients, there was one arterial thrombosis each in the apixaban arm and the dalteparin arm [21]. The single-armed interventional Cap study found a somewhat surprising 4% arterial thrombosis in patients taking apixaban, although most of those were ischemic strokes in patients with pancreatic cancer [23].

In summary, arterial thrombosis in cancer patients is only partly investigated. It would be interesting to know the frequency of arterial thrombosis in the large clinical trials on cancer-associated thrombosis. In the future, the role of prophylaxis against arterial thrombosis requires further investigation.

## 7. Conclusions

The studies on DOACs as treatment for cancer-associated VTE show that edoxaban, rivaroxaban and apixaban are treatment options for all cancer patients with VTE, but careful consideration needs to be taken regarding renal function, drug interactions, thrombocytopenia, and surgical procedures including biopsies. The risk of major bleeding in gastrointestinal cancer patients is perhaps less for apixaban than for edoxaban and rivaroxaban, especially for resected gastrointestinal cancer. There is a need for more clinical data on drug–drug interactions, but it is recommended to avoid strong inducers and inhibitors of CYP3A4 and P-glycoprotein. Incidental VTE may be false, but have a recurrence rate that is close to symptomatic VTE, and it is, therefore, reasonable to treat them the same way. Thromboprophylaxis should be considered for hospitalized cancer patients and cancer outpatients at high risk of VTE. Data on arterial thrombosis in cancer patients are starting to emerge, but there is a lack of clinical trial results.

## Figures and Tables

**Table 1:** Incidence rate of first VTE per 100 person-years by cancer type among adult patients with active cancer in UK [9].

Bladder	Breast	Colon	Lung	Prostate	Uterus	Hematologic	Brain	Ovary	Pancreas	Stomach
2.7	3.2	6.7	10	4.4	7.0	4.5	12	12	15	11

**Table 2:** Studies on cancer-associated VTE treated with vitamin K antagonists or LMWH.

Study	Design	Treatment	Treatment Length	No.	Recurrent VTE	Major Bleeding	Mortality	Metastatic Disease
Clot [13]	RCT	Coumarin	6 months	338	16%	3.6%	39%	67%
		Dalteparin		338	8.0%	5.6%		
Monreal et al. [18]	Single-armed	Dalteparin	3 months	203	8.9%	5.4%	34%	100%
Lite [14]	RCT	Warfarin	3 months	100	10%	7%	20%	42%
		Tinzaparin		100	6%	7%		
Catch [16]	RCT	Warfarin	6 months	451	10%	2.4%	32%	55%
		Tinzaparin		449	6.9%	2.7%		
Daltecan [17]	Single-armed	Dalteparin	12 months	334	11%	10%	33%	63%

RCT denotes «randomized clinical trial».

**Table 3:** Interventional studies on cancer-associated VTE treated with direct-acting oral anticoagulants or low-molecular-weight heparin.

Trial	Design	No.	Treatment	Recurrent VTE	Major Bleedings	Survival	Metastatic Disease
Hokusai [19]	RCT	1050	Dalteparin	8.8%	3.2%	76%	53%
			Edoxaban	6.5%	5.6%	73%	
Select-D [20]	RCT	406	Dalteparin	8.9%	3.0%	72%	58%
			Rivaroxaban	3.9%	5.4%	76%	
ADAM VTE [21]	RCT	287	Dalteparin	6.3%	1.4%	89%	66%
			Apixaban	0.7%	0.0%	84%	
Caravaggio [22]	RCT	1155	Dalteparin	7.9%	4.0%	74%	68%
			Apixaban	5.6%	3.8%	77%	
CAP [23]	Single-armed	298	Apixaban	4.0%	5.4%	88%	68%

RCT denotes «randomized clinical trial».

**Table 4:** Patients excluded from the clinical trials.

Study	Clinically Important Exclusion Criteria
Hokusai VTE Cancer	All other VTEs than DVT in the leg and PE. ECOG 3 and 4. 3 × upper limit normal range elevated alanine aminotransferase. Platelets <50. Life-expectancy <3 months. Treatment with NSAIDs and cox-1 and cox-2 inhibitors. Creatinine clearance below 30 mL/min.
Select-D	All other VTEs than DVT in the leg and PE. ECOG 3 and 4. Patients with primary esophageal or gastro-esophageal cancer. Patients with a previous history of VTE. Moderately elevated transaminases. Creatinine clearance below 30 mL/min.
Caravaggio	All other VTEs than proximal DVT in the leg and PE. ECOG 3 and 4. Life-expectancy <6 months. Hemoglobin level lower than 8 g/dL (5.0 mmol/L) or platelet count <75 × 10^9^/L. Moderately elevated transaminases or bilirubin. Creatinine clearance below 30 mL/min. Primary brain tumor, known intracerebral metastases, or acute leukemia.
Adam-VTE	Platelet count <50 × 10^9^/L, ALT/AST > 3 × ULN. ECOG 3 and 4. Creatinine clearance below 30 mL/min.
CAP	Creatinine clearance below 30 mL/min. Platelet count <50 × 10^9^/L Clinically significant liver disease.

**Table 5:** Numbers of major bleedings during 6 months treatment in patients with gastrointestinal and genitourinary cancers.

Study ***	Treatment	Gastrointestinal Cancer	Genitourinary Cancer	Other Cancers
Hokusai * [24]	Edoxaban	21/165 (12.7%)	3/65 (4.6%)	8/292 (2.7%)
	Dalteparin	5/140 (3.6%)	1/71 (1.4%)	13/313 (4.2%)
Select-D ** [20]	Rivaroxaban	8/91 (8.8%)	1/25 (4%)	2/87 (2.3%)
	Dalteparin	5/86 (5.8%)	0/17 (0%)	1/100 (1.0%)
Caravaggio [25]	Apixaban	9/188 (4.8%)	4/66 (6.0%)	9/322 (2.8%)
	Dalteparin	9/187 (4.8%)	6/73 (8.2%)	8/319 (2.5%)
CAP [23]	Apixaban	7/126 (5.5%)	3/55 (5.4%)	5/117 (4.3%)

* 12 months data. ** Patients with cancer of the esophagus or gastroesophageal junction were excluded after 220 of 406 included patients. *** Adam-VTE study had no major bleedings in the apixaban-arm.

**Table 6:** Recurrent VTE based on symptomatic or incidental index VTE.

Trial *	Number of Patients	Type of Index VTE	Recurrent VTE	
			DOAC	Dalteparin
Hokusai	1050	Symptomatic: 706	9.3%	11.7%
		Incidental: 340	4.8%	10.4%
Caravaggio	1155	Symptomatic: 925	6.3%	8.4%
		Incidental: 230	2.6%	6.1%
Cap	298	Symptomatic: 136	3.6%	n.a.
		Incidental: 162	4.3%	

* The Adam-VTE and Select-D trial did not report detailed recurrent VTE numbers based on symptomatic/incidental index VTE.

**Table 7:** Examples of drugs interacting with DOACs.

Strong Inhibitors of CYP3A4 or P-Glycoprotein	
Antifungal drugs	Voriconazole, Itraconazole, Posaconazole, (Fluconazole?)
Cancer drugs	Idelalisib
Drugs against HIV and Hepatitis C	Ritonavir, Indinavir, Nelfinavir, Sakinavir, Darunavi, Simeprevir, Boceprevir, Telaprevir, Kobicistat
**Strong Inducers of CYP3A4 or P-Glycoprotein**	
Antiepileptic drugs	Phenytoin, Carbamazepine, Phenobarbital, Primidone
Cancer drugs	Enzalutamide, Dabrafenib
Other drugs	Rifampicin, St. John’s wort, Efavirenz

**Table 8:** Studies on pharmacological thromboprophylaxis for VTE in cancer patients.

Study (Drug)	VTE Placebo	VTE Drug	Major Bleeding on Drug	NNT *
Protecht [36] (nadroparin)	3.1%	1.6%	0.7%	67
Save-Onco [29] (semuloparin)	3.4%	1.2%	1.2%	45
Avert [37] (apixaban)	10.2%	4.2%	3.5%	17
Cassini [38] (rivaroxaban)	8.8%	6.0%	2.0%	36

* NNT refers to the number of patients needed to treat to prevent one VTE.

**Table 9:** Arterial thrombosis in cancer patients receiving anticoagulation.

Study	Indication	Treatment	Arterial Thrombosis
Protecht [36]	Prophylaxis	Nadroparin	3/769 (0.4%)
		Placebo	3/381 (0.8%)
Cassini [38]	Prophylaxis	Rivaroxaban	4/420 (1.0%)
		Placebo	7/421 (1.7%)
Adam-VTE [21]	Treatment	Apixaban	1/145 (0.7%)
		Dalteparin	1/142 (0.7%)
Cap [23]	Treatment	Apixaban	12/298 (4.0%)
